# Insulin resistance enhances the mitogen-activated protein kinase signaling pathway in ovarian granulosa cells

**DOI:** 10.1371/journal.pone.0188029

**Published:** 2017-11-10

**Authors:** Linghui Kong, Qien Wang, Jiewen Jin, Zou Xiang, Taoyu Chen, Shanmei Shen, Hongwei Wang, Qian Gao, Yong Wang

**Affiliations:** 1 State Key Laboratory of Analytical Chemistry for Life Science & Jiangsu Key Laboratory of Molecular Medicine, Medical School, Nanjing University, Nanjing, Jiangsu, China; 2 Department of Health Technology and Informatics, Faculty of Health and Social Sciences, The Hong Kong Polytechnic University, Hong Kong, China; 3 Divisions of Endocrinology, The Affiliated Drum Tower Hospital, Medical School, Nanjing University, Nanjing, Jiangsu, China; Peking University Third Hospital, CHINA

## Abstract

The ovary is the main regulator of female fertility. Granulosa cell dysfunction may be involved in various reproductive endocrine disorders. Here we investigated the effect of insulin resistance on the metabolism and function of ovarian granulosa cells, and dissected the functional status of the mitogen-activated protein kinase signaling pathway in these cells. Our data showed that dexamethasone-induced insulin resistance in mouse granulosa cells reduced insulin sensitivity, accompanied with an increase in phosphorylation of p44/42 mitogen-activated protein kinase. Furthermore, up-regulation of cytochrome P450 subfamily 17 and testosterone and down-regulation of progesterone were observed in insulin-resistant mouse granulosa cells. Inhibition of p44/42 mitogen-activated protein kinase after induction of insulin resistance in mouse granulosa cells decreased phosphorylation of p44/42 mitogen-activated protein kinase, downregulated cytochrome P450 subfamily 17 and lowered progesterone production. This insulin resistance cell model can successfully demonstrate certain mechanisms such as hyperandrogenism, which may inspire a new strategy for treating reproductive endocrine disorders by regulating cell signaling pathways.

## Introduction

The granulosa cell (GC), the oocytes’ companion cell, ignites a continuous cross-talk between the somatic and germ cell compartments [1]. GC dysfunction may be involved in various reproductive endocrine disorders including Cushing’s syndrome [2], polycystic ovary syndrome (PCOS) [3] and premature ovarian failure [4]. Abnormal glucose metabolism is an important type of GC dysfunction, in which insulin resistance (IR) plays a significant role [5]. Therefore, elucidation of the cellular IR model is critical in understanding the pathological changes in dysfunctional GCs from both clinical and basic medical science perspectives.

Dexamethasone (Dex) is a classical glucocorticoid, which triggers unbalanced cellular glucose metabolism, interferes with insulin sensitivity and induces IR by enhancing the function of pancreatic islet β cells, resulting in the inhibition of glucose uptake [6–8]. Therefore, Dex is often employed to model cell IR. Dex-induced IR can mimic various disorders such as Cushing’s syndrome [9] and PCOS [10]. Female patients with Cushing's syndrome usually manifest gonadal dysfunction, menstrual disorders and secondary amenorrhea, as well as acne, hirsutism and even virilization [2, 11–13]. PCOS is a common endocrine disorder of reproductive-aged women [14, 15] exhibiting main clinical features of menstrual disorder, hyperandrogenism and polycystic ovary [16–19].

Conventional mitogen-activated protein kinases (MAPKs) include p44/42 MAPK, c-Jun N-terminal kinase (JNK) and p38 MAPK [20]. The p44/42 MAPK, which is the focus of our study, is regulated by upstream RAS/RAF as an activator protein. RAS/RAF activates the MAPK kinase (MAPKK, MEK), which phosphorylates downstream p44/42 MAPK after phosphorylation [21]. Phosphorylated p44/42 MAPK has the ability to enter nucleus and promote the expression of signal transducer and activator of transcription 3 (STAT3) and c-fos [22]. MAPK signaling plays a crucial role in human growth and development. The MAPK signaling pathway is also involved in IR [23]. Therefore, the inhibition of the p44/42 MAPK signaling pathway may have an effect on IR. PD98059 is a specific inhibitor of the p44/42 MAPK signaling pathway, which can inhibit phosphorylation of MEK, MAPK, and the downstream molecules, therefore altering the regulatory roles of the MAPK signaling pathway. A recent study showed that PD98059 decreases phosphor-MAPK in ovarian theca cells [24].

In this study, we focused on the GC-based IR model and investigated the role of the p44/42 MAPK signaling pathway in this model. Our model may illustrate possible mechanisms of IR in GCs, and provide further insight into the possible therapeutic development of reproductive endocrine disorders.

## Materials and methods

### Isolation and culture of primary mouse GCs

3-week-old female wild-type Institute of Cancer Research (ICR) mice (Model Animal Research Center of Nanjing University, Nanjing, China) were injected with pregnant mare serum gonadotropin (PMSG) (Ningbo Sansheng Pharmaceutical, Ningbo, China) (10 U/10 g weight) 48 h before sacrifice. Mice were housed in separate cages under 21°C with a 12 h light/12 h dark cycle. Free access to a standard diet and water supply was provided. Mice were killed by cervical dislocation. Next, killed mice were soaked with 75% ethanol (Nanjing Chemical Reagent, Nanjing, China) for 15 min followed by removal of bilateral ovaries. Ovaries were soaked with 10 ml Dulbecco's Modified Eagle's medium (DMEM)-Ham's F12 nutrient medium (F12) with 2% penicillin (10, 000 U/ml)-streptomycin (Pen-Strep) (10,000 μg/ml) (Thermo Fisher Scientific, Waltham, USA) in a petri dish after removal of the surrounding adipose tissue and cleaning of bloodstains. Follicles were separated and punctured by micro-forceps to release GCs under a microscope, and the remaining ovarian theca tissue was discarded. GC suspension was transferred to a centrifuge and was centrifuged at 1000 rpm for 5 min. Next, supernatant was discarded and GCs were suspended in DMEM-F12 with 10% fetal bovine serum (FBS) (Thermo Fisher Scientific) and 1% Pen-Strep (culture media), and then transferred to a 25 ml cell culture flask and placed in an incubator at 37°C, 5% carbon dioxide [25]. Each flask contains GCs extracted from 3 mice. Cells were sub-cultured after reaching confluence in the 6-well plates or 24-well plates. Animal research has been approved by the Drum Tower Hospital ethics committee. An ethnics statement chart has been provided to the editors.

### Establishment of the IR models of GC and inhibition of the MAPK signaling pathway

As IR correlates to glucose intolerance and metabolic disorders [26], measuring glucose concentrations is important to identify IR. To determine whether the IR models of GC were successfully established, we divided the GCs cultured in 24-well plates into 4 groups. The control group (Con) did not receive any treatment; the Dex group (GD) was treated with Dex (Cisen Pharmaceutical, Jining, China.) (100 μmol/L) for 48 h before harvesting supernatant; the insulin group (GI) was treated with human insulin (Jiangsu Wanbang Biopharmaceuticals, Xuzhou, China) (5 IU) [27] for 1 h before harvesting supernatant; the Dex + insulin group (GDI) was treated with Dex (100 μmol/L) for 48 h and insulin (5 IU) was added 1 h prior to the end of the culture. To further investigate the effect of IR either in the absence or presence of inhibition of MAPK signaling pathway on GCs, we divided the GCs cultured in 6-well plates or 24-well plates into 3 groups: Con, GD as described above and the Dex + PD98059 group (GDP) which was treated with Dex (100 μmol/L) for 48 h and PD98059 (Sigma-Aldrich, Steinheim, Germany) at 50 μmol/L was added 4 h before the end of the culture [28].

### Measurement of glucose in GC culture supernatants

Culture media were collected and centrifuged at 1000 × g for 15 min. Supernatants were collected for measuring glucose concentrations with the Glucose Oxidase Method assay kit (Applygen Technologies, Beijing, China). Briefly, 5 μl sample was incubated with 295 μl regent consisting of two components from the kit (R1:R2 = 4:1) for 20 minutes in 37°C. The OD values at 570 nm were measured to determine glucose concentrations of the samples.

### Measurement of the proliferative activity of GCs

GC proliferative activity was measured by the methylthiazolyldiphenyl-tetrazolium bromide (MTT) cell proliferation and cytotoxicity assay kit (Beyotime Biotechnology, Shanghai, China).

### Protein extraction and western blot analysis

Cells were incubated in the radio-immunoprecipitation assay (RIPA) lysis buffer (Beyotime Biotechnology) for 30 min on ice for complete lysis. For every 10 ml RIPA buffer, one phosphatase inhibitor mini tablet (Thermo Fisher) was added. The lysate was centrifuged at 13,000 rpm for 15 min at 4°C for harvesting the total protein which was denatured at 95°C for 10 min. Protein concentration was measured by a BCA protein assay kit (Thermo Fisher). Equal amounts (20 μg) of total protein were separated by 10% sodium dodecyl sulfate polyacrylamide gel electrophoresis (SDS-PAGE) followed by transferring to a polyvinylidenediflouride membrane (Merck Millipore, Steinheim, Germany) which was blocked with 5% albumin bovine V (Amresco, Solon, USA). The membrane was incubated with a specific antibody overnight at 4°C [29]. Antibodies were diluted as follows: Anti-β-actin rabbit monoclonal antibody (mAb) (1: 5,000, Bioworld Technology, St. Louis, USA); anti-p44/42 MAPK rabbit mAb (1: 1,000); anti-phospho-p44/42 MAPK rabbit mAb (1: 2,000); anti-JNK rabbit mAb (1: 1,000); anti-phospho-JNK rabbit mAb (1: 1,000); anti-p38 MAPK rabbit mAb (1: 1,000); anti-phospho-p38 MAPK rabbit mAb (1: 1,000); anti-protein kinase B (Akt) rabbit mAb (1: 1,000); anti-phospho Akt rabbit mAb (1: 2,000); anti-STAT3 rabbit mAb (1: 2,000) and anti-phospho-STAT3 rabbit mAb (1: 2,000) (Cell Signal Technology, Denver, USA); anti-cytochrome P450 subfamily 17 (CYP17) rabbit antibody (1: 1,000, Abcam, Cambridge, U.K.); anti-aromatase rabbit antibody (1: 200, Santa Cruz Biotechnology, Dallas, USA). Subsequently, the membranes were incubated with horse radish peroxidase (HRP)-linked goat anti rabbit immunoglobulin G (IgG) (1: 10,000, Bioworld Technology) followed by detection using the immobilon western chemiluminescent HRP substrate (Merck Millipore) [25]. β-actin was used to normalize the concentrations of the target protein.

### Analysis of hormone production

Estradiol (E2), testosterone (T) and progesterone (P4) were measured by enzyme-linked immunosorbent assay (ELISA) (Elabscience Biotechnology, Wuhan, China).

### Statistical analysis

Data are shown as the mean + standard error of mean (SEM) and were analyzed with Graphpad prism 5.01 (Graphpad Inc.; La Jolla, USA) for statistical significance using one-way analysis of variance (ANOVA) among groups. A p value of 0.05 or less is considered statistically significant.

## Results

### Dex decreases the sensitivity of GCs to insulin

**Fig 1:**
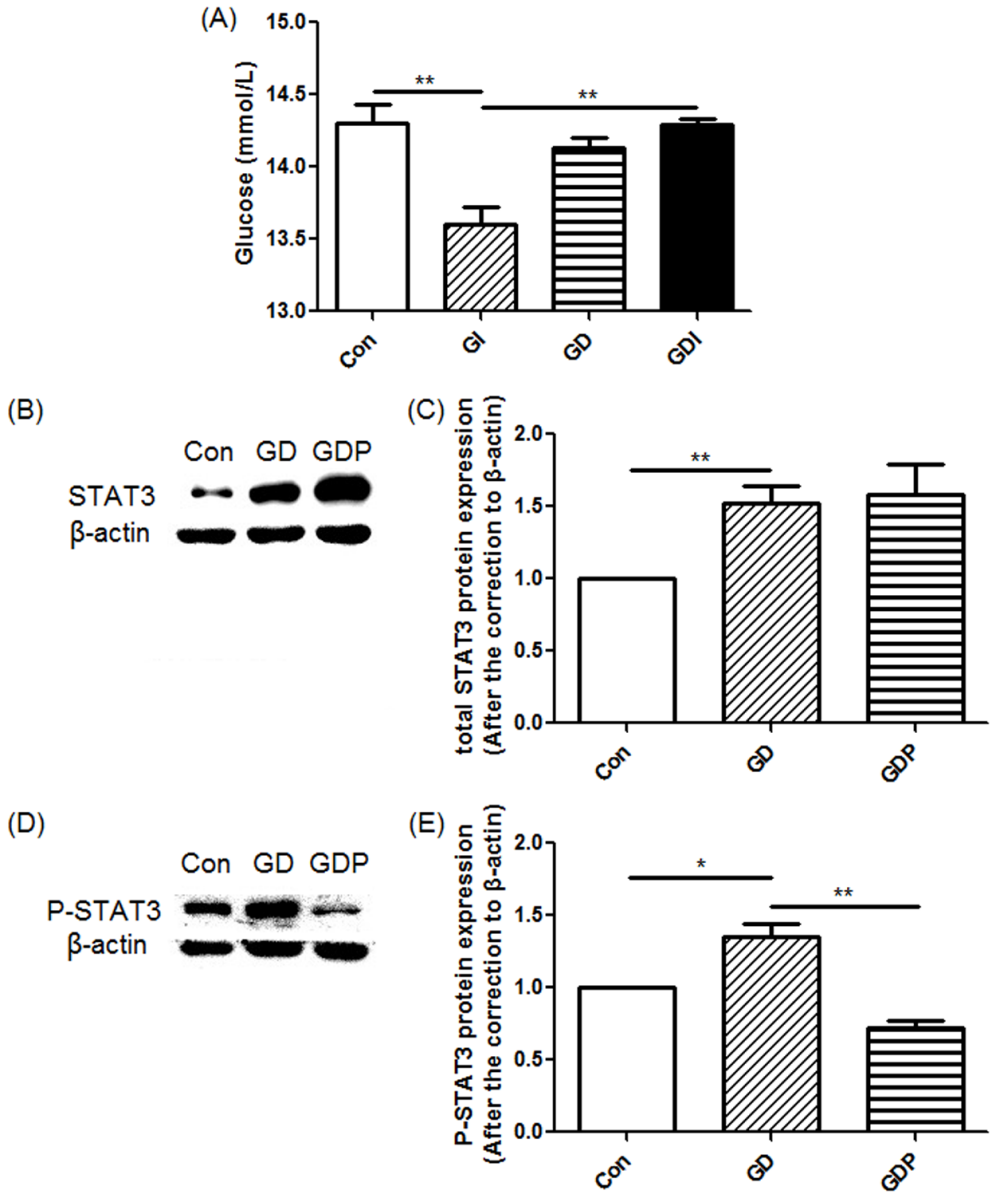
Glucose concentrations, signal transducer and activator of transcription 3 (STAT3) and phosphor-STAT3 (P-STAT3) protein expression in granulosa cells (GCs). (A) GCs were incubated in the absence (Con) or presence of dexamethasone (Dex) for 48 h (GD), insulin (GI) for 1 h, or Dex for 48 h with insulin added 1 h before the end of the incubation (GDI). (B-E) GCs were incubated in the absence (Con) or presence of Dex for 48 h (GD) or Dex for 48 h with PD98059 added 4 h before the end of the incubation (GDP). Relative density ratios were calculated by setting the control group value as one. Each bar represents the mean + SEM. All data presented are representative of at least three separate experiments. *p < 0.05, **p < 0.01.

The most important characteristic of IR is the decrease in insulin sensitivity [26]. We first determined GC insulin sensitivity by measuring the glucose concentrations in the supernatant of GC culture. As shown in Fig 1A, presence of insulin resulted in a decrease of glucose concentration in the supernatant of GC culture (p = 0.004), which indicated that insulin is capable of promoting glucose utilization. Nevertheless, treatment with Dex completely inhibited insulin-mediated reduction in glucose concentration (p = 0.001), implicating that Dex can counteract the effect of insulin. Treatment with Dex alone did not regulated glucose concentration (p = 0.289), suggesting that Dex could not affect glucose concentration by itself but was able to interfere with the glucose-regulatory function of insulin. Our data demonstrated that IR could be induced by treatment with Dex.

To further demonstrate the effect of Dex on IR, We measured Dex-mediated regulation of STAT3 and phosphor-STAT3 as elevated STAT3 phosphorylation is suggested to correlate to IR [30–33]. Total STAT3 (p = 0.003) and phospho-STAT3 (p = 0.029) were both elevated in GCs following treatment with Dex (Fig 1B–1E), supporting that Dex can induce IR in GCs.

Previous studies demonstrated the relations between IR and the downregulation of the phosphatidylinositol 3 kinase (PI3K)/Akt signaling pathway [34, 35]. Hence, we measured Akt and phosphor-Akt to validate our GC-based IR model. Our results showed that Dex inhibited Akt phosphorylation (p = 0.013) without affecting total Akt (p = 0.079) (S1 Fig), which is consistent with literature reports. Phosphor-Akt was not alerted after the addition of PD98059 in the GC IR model (p = 0.133) (S1 Fig).

### Inhibition of the p44/42 MAPK signaling suppresses the Dex-induced up-regulation of phosphor-p44/42 MAPK

**Fig 2:**
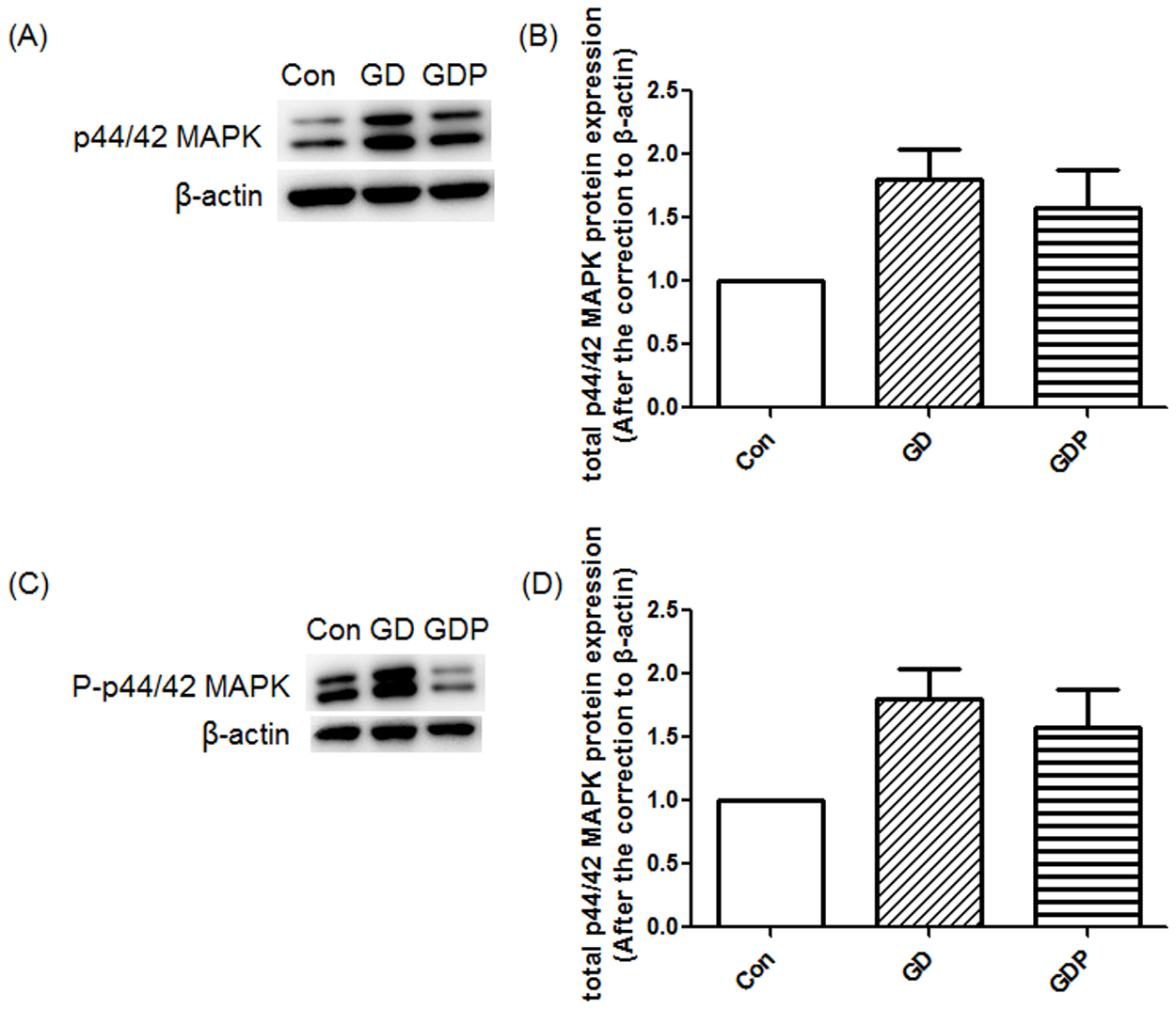
Mitogen-activated protein kinase (MAPK) and phosphor-p44/42 MAPK (P-MAPK) protein expression in GCs. GCs were incubated in the absence (Con) or presence of Dex for 48 h (GD) or Dex for 48 h with PD98059 added 4 h before the end of the incubation (GDP). Relative density ratios were calculated by setting the control group value as one. Data are expressed as the mean + SEM. All data presented are representative of at least three separate experiments. *p < 0.05, ***p < 0.001.

As a fundamental downstream pathway of the insulin receptor, the p44/42 MAPK signaling pathway correlates with IR [23]. We therefore measured the concentrations of p44/42 MAPK and phosphor-p44/42 MAPK. Dex elevated phosphor-p44/42 MAPK (p = 0.012), without affecting total p44/42 MAPK (p = 0.058), which demonstrated that Dex could enhance the p44/42 MAPK signaling pathway. Furthermore, GCs treated with both Dex and PD98059 significantly suppressed Dex-induced up-regulation of phosphor-p44/42 MAPK (p < 0.001) (Fig 2).

Furthermore, we investigated the other two signaling molecules of the conventional MAPK pathways, JNK and p38 MAPK. Dex elevated phosphor-JNK (p = 0.011) and total p38 MAPK (p = 0.020), which demonstrates that the key molecules on these two routes of the conventional MAPK signaling pathway might be increased in our GC-based IR model. In addition, we observed further increase of phosphor-JNK after the addition of PD98059. We hypothesize that inhibition of the p44/42 MAPK signaling pathway leads to a compensatory increase in the JNK signaling pathway (S2 Fig).

### Dex decreases the GC proliferative activity

**Fig 3:**
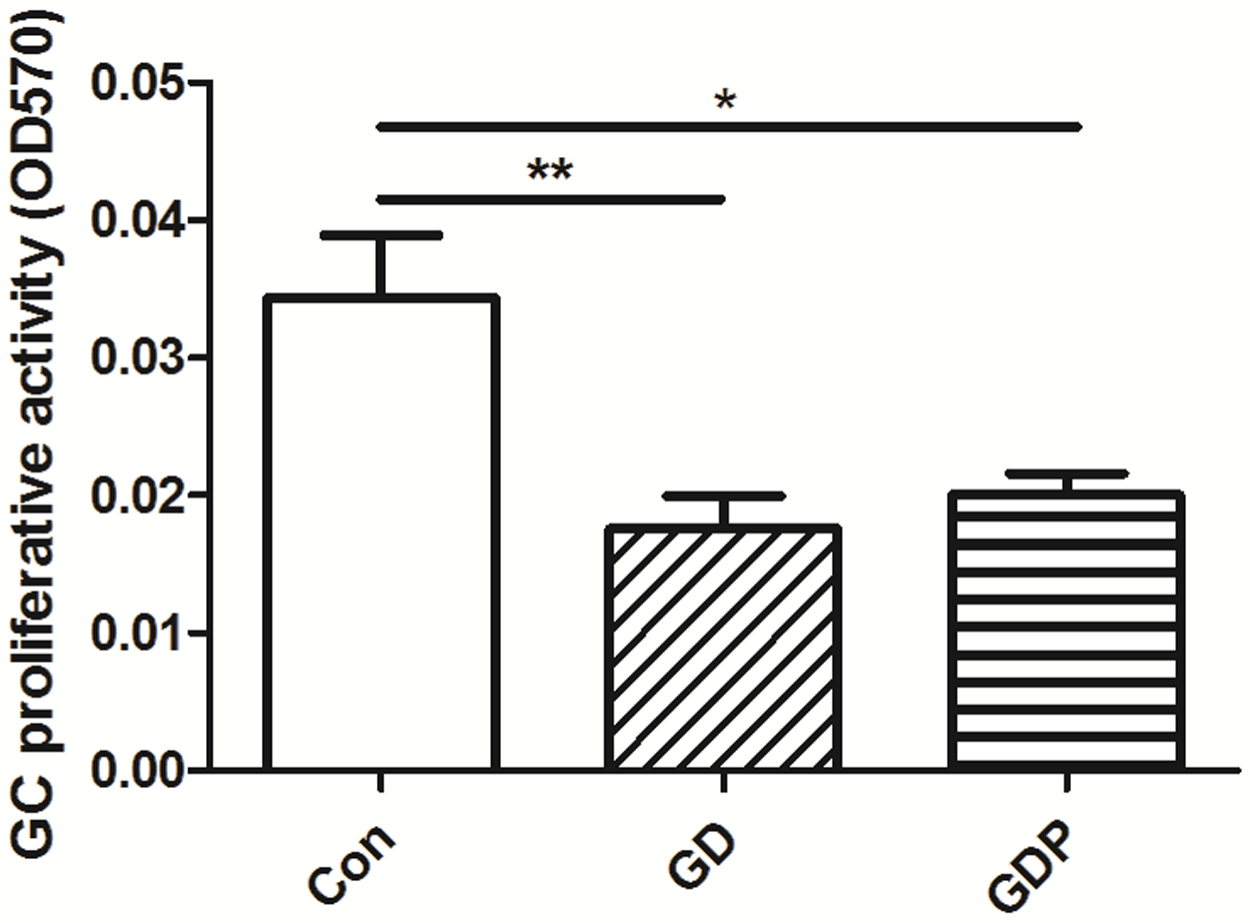
Effects of Dex and PD98059 on GC proliferative activity. GCs were incubated in the absence (Con) or presence of Dex for 48 h (GD) or Dex for 48 h with PD98059 added 4 h before the end of the incubation (GDP). GC proliferative activity was measured by the methylthiazolyldiphenyl-tetrazolium bromide (MTT) method. Data are expressed as the mean + SEM. All data presented are representative of at least three separate experiments. *p < 0.05, **p < 0.01.

Dex shares the common side effect such as metabolic disorders with other glucocorticoids [8]. Furthermore, as Dex can alter the activity of the MAPK signaling pathway, the cytotoxic effect of Dex needs to be clarified. To determine whether Dex and PD98059 have an impact on GC proliferative activity, we undertook to detect the proliferative activity of GCs. Our results showed that both Dex (p = 0.005) and PD98059 (p = 0.013) suppressed the proliferation of GCs (Fig 3). This experiment implicated that Dex may exhibit a cytotoxic effect on GCs.

### PD98059 suppresses Dex-induced up-regulation of CYP17 protein expression in GCs

**Fig 4:**
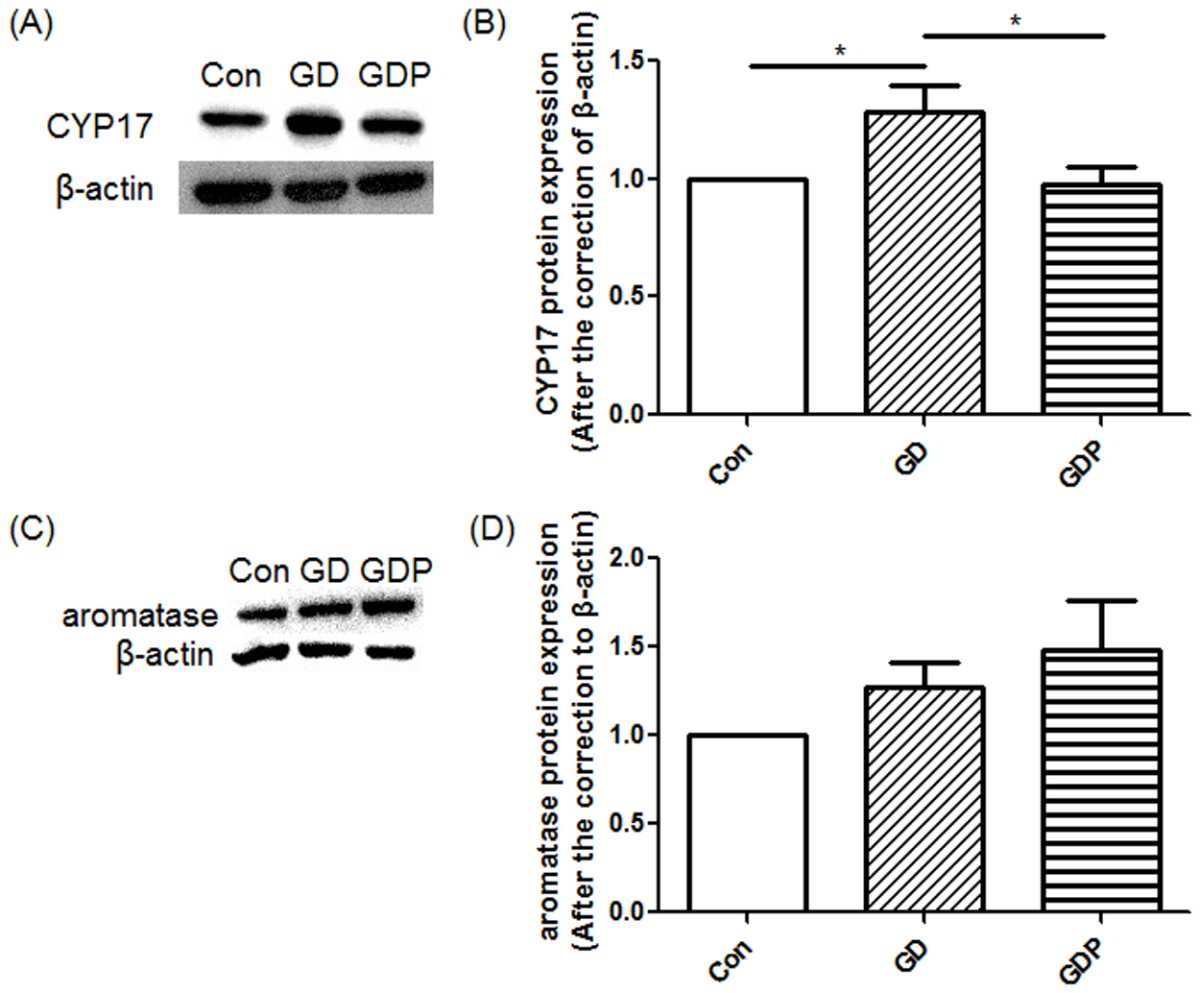
Cytochrome P450 subfamily 17 (CYP17) and aromatase protein expression in GCs. GCs were incubated in the absence (Con) or presence of Dex for 48 h (GD) or Dex for 48 h with PD98059 added 4 h before the end of the incubation (GDP). Relative density ratios were calculated by setting the control group value as one. Data are expressed as the mean + SEM. All data presented are representative examples of at least three separate experiments. *p < 0.05.

According to the two-cell-two-gonadotropin theory, aromatase can transform T into E2 in GCs [36], which may regulate the concentrations of related hormones. On the other hand, in ovarian theca cells, CYP17, along with several other enzymes, can transform cholesterol into DHEA and T [36], which indicates that CYP17 is the key enzyme of the synthesis of androgen. Yet a human GC line (KGN) can also express CYP17 [37], suggesting that GCs may also produce DHEA and T. To delineate the role of the MAPK signaling pathway in GC steroidogenesis, we cultured the cells in the presence or absence of Dex and PD98059. GCs treated with only Dex resulted in significant increases in CYP17 (p = 0.026), while GCs treated with Dex and PD98059 significantly suppressed Dex-induced up-regulation of CYP17 (p = 0.033) (Fig 4A and 4B), supporting that the MAPK signaling pathway may play a crucial role in the regulation of androgen secretion. On the other hand, Dex exhibited no influence on aromatase in either the presence or absence of PD98059 (p = 0.228) (Fig 4C and 4D), which may indicate that aromatase is not regulated by the MAPK signaling pathway. Furthermore, PD98059 significantly suppressed Dex-induced up-regulation of phosphor-STAT3 in GCs (p = 0.003) (Fig 1D and 1E), supporting that IR can be improved by inhibiting the MAPK signaling pathway, as elevated STAT3 phosphorylation is considered correlated to IR [30–33].

### Dex-induced IR increases the T production and decreases P4 production in GCs

**Fig 5:**
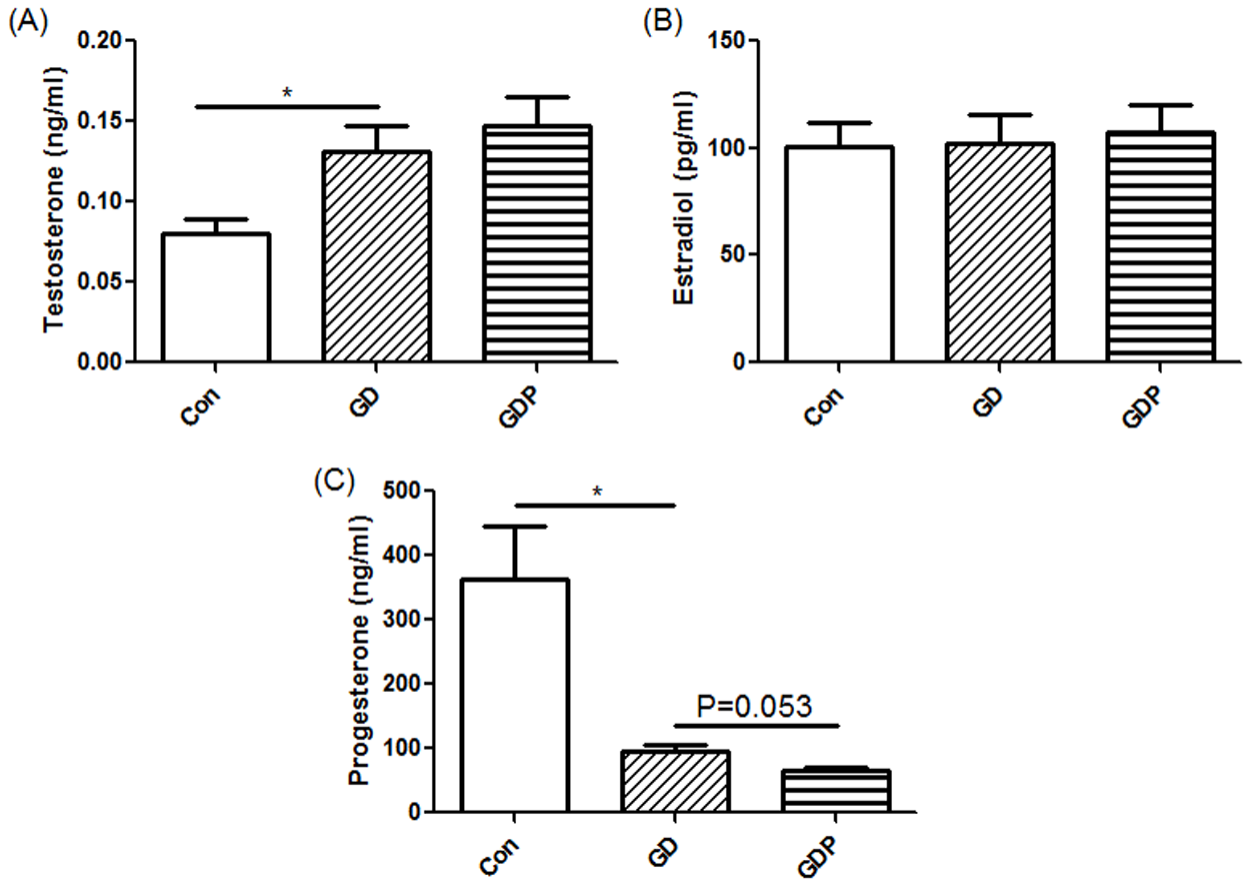
Testosterone (T), estradiol (E2) and progesterone (P4) production by GCs. GCs were incubated in the absence (Con) or presence of Dex for 48 h (GD) or Dex for 48 h with PD98059 added 4 h before the end of the incubation (GDP). Data presented are representative of at least three separate experiments. *p < 0.05.

In this study, the steroidogenic activities of GCs were evaluated. The concentrations of T, E2 and P4 secreted from GCs treated for 4 h with Dex in the presence or absence of PD98059 were measured, as these hormones are important in the female endocrine system [19, 38, 39]. Basal secretion of T, E2 and P4 from GCs was detectable in the medium. In contrast, T was significantly increased after incubation of the cells with Dex (p = 0.031), and such increase was not modulated by PD98059 (p = 0.543) (Fig 5A), demonstrating that hyperandrogenism existed in the GC IR model. However, hyperandrogenism could not be regulated by suppressing the MAPK signaling pathway. Neither Dex alone nor Dex in the presence of PD98059 modulated E2 production (p = 0.936) (Fig 5B), which is consistent with the regulation of aromatase in the same setting. Interestingly, in contrast, P4 was significantly decreased after incubation of the cells with Dex (p = 0.048). Dex together with PD98059 demonstrated a trend of being able to further down-regulate P4 (p = 0.053) (Fig 5C).

## Discussion

In this research, we undertook to uncover the pathological changes in dysfunctional GCs by creating an IR cell model and shed light on the importance of the inhibition of the p44/42 MAPK signaling pathway in the GC IR model, which may be achieved through regulating CYP17 and androgen expression. The IR cell model can successfully demonstrate certain mechanisms such as hyperandrogenism, which may inspire a new strategy for treating reproductive endocrine disorders through regulating the cell signaling pathways.

Theoretically, ovarian theca cells are required to produce T, and GC is expected to convert T into E2 [36]. Therefore, CYP17 expression has minor effects on altering the T concentration in culture despite the fact that it is observed in GCs. On the other hand, since T participates in E2 production in GCs, the consumption of T will also influence E2 concentration in culture. The two aspects aforementioned may explain our observation that the concentration of T was elevated in the GC IR model. On top of that, it is reported that P4 production only occurs in luteinized GCs [40–42]. We observed P4 production in ovarian GCs in our experiment, which implies that luteinization possibly occurs in our GCs. It took several days to cultivate our cells, which may induce the cells to luteinize partially. Nevertheless, previous research showed that E2 production also occurs in luteinizing hormone (LH)-induced luteinized GCs [42, 43], illustrating that T consumption also occurs in luteinized GCs, which can further effect the concentration of T in the GC culture.

Previous studies reported that in human HepG2 cells and rat cardiomyocytes, the p44/42 MAPK signaling pathway is up-regulated after IR [44, 45], which confirmed our data from a different perspective. Nevertheless, aother study showed that the p44/42 MAPK signaling pathway is down-regulated in the PCOS-related GC IR models [46], which is inconsistent with our data. In PCOS patients, the IR phenotype appears in parallel with hyperinsulinemia, a process similar to type 2 diabetes. In the studies aforementioned, the cell culture was supplied with extra insulin in order to mimic a hyperinsulinemic environment. This may largely account for the reduction in p44/42 MAPK phosphorylation. In our experiments, no exogenous insulin was added into the cell culture, which may be the difference between our GC IR model and the PCOS GCs in the literature described above.

A study has shown that IR activates conventional MAPKs, including p44/42 MAPK, JNK and p38 MAPK [35]. Our research witnessed phosphor-p44/42 MAPK and phosphor-JNK upregulation, while phosphor-p38 MAPK was not significantly upregulated. However, another study on adipocytes revealed that Dex-induced IR down regulates phosphor-p38 MAPK [47]. These two studies elicit controversy on the relations between IR and the p38 MAPK signaling pathway, which requires further research for clarification.

Besides the MAPK signaling pathway, the PI3K/Akt signaling pathway is another important signaling pathway down-regulated by the insulin receptor [48]. It is reported that the alteration of the PI3K-glucose transporter type 4 (GLUT4) signaling pathway can reverse IR and decrease blood glucose concentrations in rats [49], which demonstrated that the PI3K/Akt signaling pathway is responsible for the regulation of IR. Furthermore, the PI3K/Akt signaling pathway is correlated with CYP17 [50], and therefore may possibly regulate the concentration of androgen. Our study witnessed down regulation of phosphor-Akt in our GC IR model, yet we observed no significant differences for Akt phosphorylation after suppressing the p44/42 MAPK signaling pathway. In our work, Dex-mediated up-regulation of CYP17, but not T, was reversed by suppressing the p44/42 MAPK signaling pathway. We speculate that the PI3K/Akt signaling pathway may play a role in this differential regulatory process, which may be elucidated in future studies.

## Conclusion

Our data confirmed that in the GC IR model, CYP17 is elevated as a result of the up-regulation of the p44/42 MAPK signaling pathway, which may facilitate T elevation. Inhibition of the p44/42 MAPK signaling pathway can down-regulate CYP17. Nevertheless, whether the concentration of androgen can be down-regulated by inhibiting the MAPK signaling pathway needs to be further clarified. IR can reduce the P4 concentration owing to the loss of GC viability. On the other hand, the concentration of P4 was further decreased after suppressing the p44/42 MAPK signaling pathway (S3 Fig). Taken together, our work reveals a possible mechanism of the MAPK signaling pathway regulation in the GC IR model.

## Supporting information

S1 Fig Protein kinase B (Akt) and phosphor-Akt (P-Akt) protein expression in GCs. GCs were incubated in the absence (Con) or presence of Dex for 48 h (GD) or Dex for 48 h with PD98059 added 4 h before the end of the incubation (GDP). Relative density ratios were calculated by setting the control group value as one. Data are expressed as the mean + SEM. All data presented are representative of at least three separate experiments. *p < 0.05.
(TIF)

S2 Fig c-Jun N-terminal kinase (JNK), phosphor-JNK (P-JNK), p38 mitogen-activated protein kinase (MAPK) and phosphor-p38 MAPK (P-p38 MAPK) protein expression in GCs. GCs were incubated in the absence (Con) or presence of Dex for 48 h (GD) or Dex for 48 h with PD98059 added 4 h before the end of the incubation (GDP). Relative density ratios were calculated by setting the control group value as one. Data are expressed as the mean + SEM. All data presented are representative of at least three separate experiments. *p < 0.05.
(TIF)

S3 Fig Diagram representing a putative mechanistic model of the p44/42 MAPK signaling pathway in the GC insulin resistance (IR) model. For normal GCs, P4 is converted from cholesterol followed by partial conversion into T through catalyzation of CYP17. External T is transferred into GC and converted into E2 along with internal T, which is later secreted through catalyzation of aromatase. For the GC IR model, up regulation of the p44/42 MAPK signaling pathway leads to the elevation of the CYP17 level while reducing T intake, resulting in an increased concentration of T. Inhibition of the p44/42 MAPK signaling pathway by PD98059 results in the down-regulation of CYP17.
(TIF)
