# Correction: Insulin resistance enhances the mitogen-activated protein kinase signaling pathway in ovarian granulosa cells

**DOI:** 10.1371/journal.pone.0249806

**Published:** 2021-04-05

**Authors:** Linghui Kong, Qien Wang, Jiewen Jin, Zou Xiang, Taoyu Chen, Shanmei Shen, Hongwei Wang, Qian Gao, Yong Wang

Fig 2D is incorrect. The authors have provided a corrected version of Fig 2 here.

**Fig 2:**
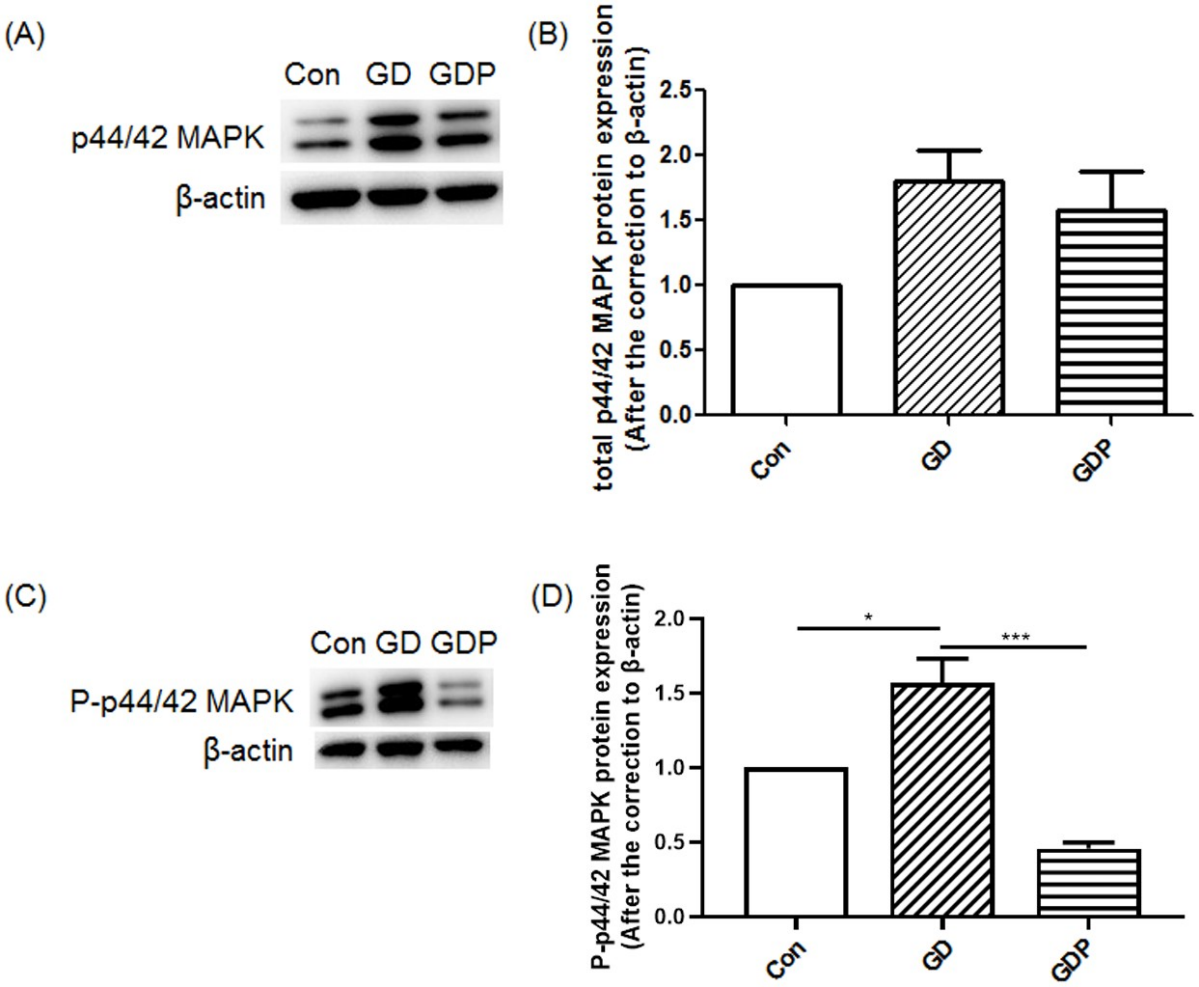
Mitogen-activated protein kinase (MAPK) and phosphor-p44/42 MAPK (P-MAPK) protein expression in GCs. GCs were incubated in the absence (Con) or presence of Dex for 48 h (GD) or Dex for 48 h with PD98059 added 4 h before the end of the incubation (GDP). Relative density ratios were calculated by setting the control group value as one. Data are expressed as the mean + SEM. All data presented are representative of at least three separate experiments. *p < 0.05, ***p < 0.001.
