# Supplementary figures and images for: Insulin resistance enhances the mitogen-activated protein kinase signaling pathway in ovarian granulosa cells
Source: PLoS One. 2017 Nov 10;12(11):e0188029. doi: 10.1371/journal.pone.0188029 (PMC5695281; doi:10.1371/journal.pone.0188029)

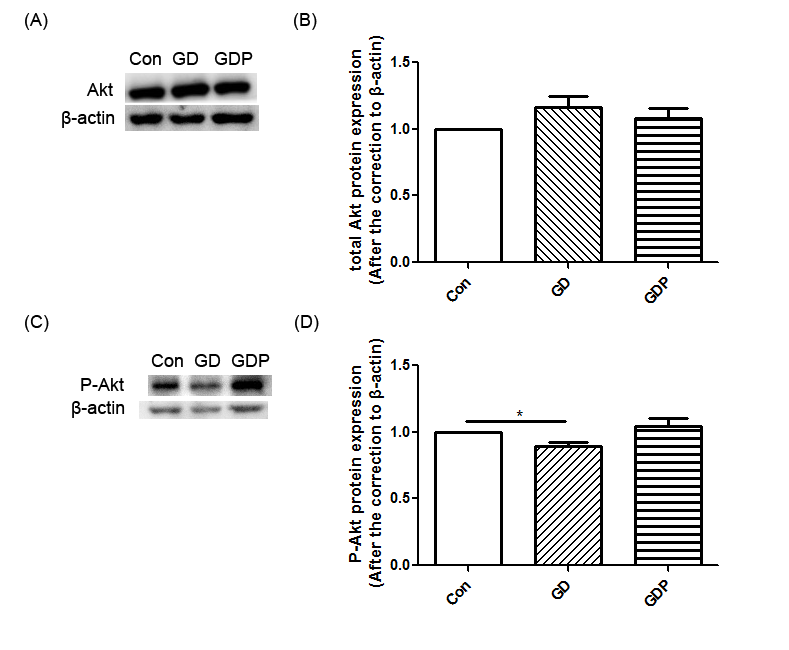

Supplement: S1 Fig — GCs were incubated in the absence (Con) or presence of Dex for 48 h (GD) or Dex for 48 h with PD98059 added 4 h before the end of the incubation (GDP). Relative density ratios were calculated by setting the control group value as one. Data are expressed as the mean + SEM. All data presented are representative of at least three separate experiments. *p < 0.05. (TIF) [file pone.0188029.s001.tif]

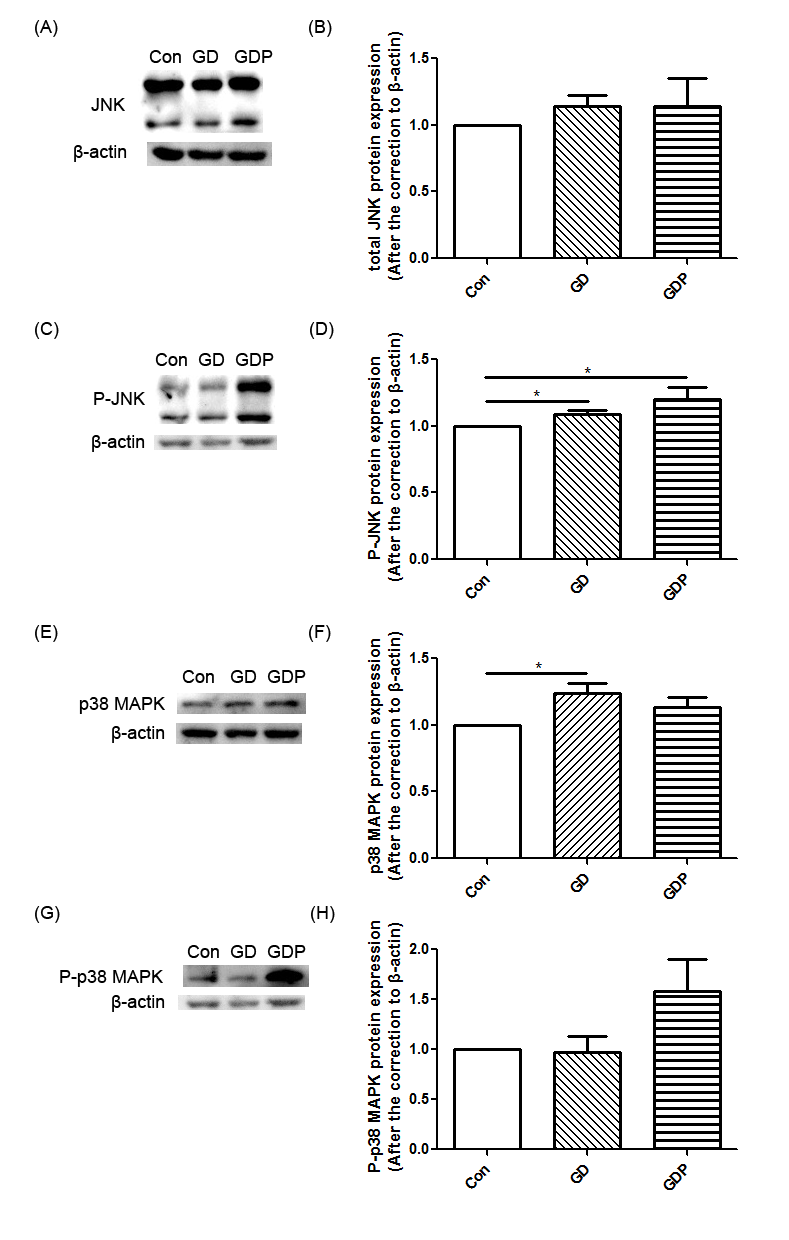

Supplement: S2 Fig — GCs were incubated in the absence (Con) or presence of Dex for 48 h (GD) or Dex for 48 h with PD98059 added 4 h before the end of the incubation (GDP). Relative density ratios were calculated by setting the control group value as one. Data are expressed as the mean + SEM. All data presented are representative of at least three separate experiments. *p < 0.05. (TIF) [file pone.0188029.s002.tif]

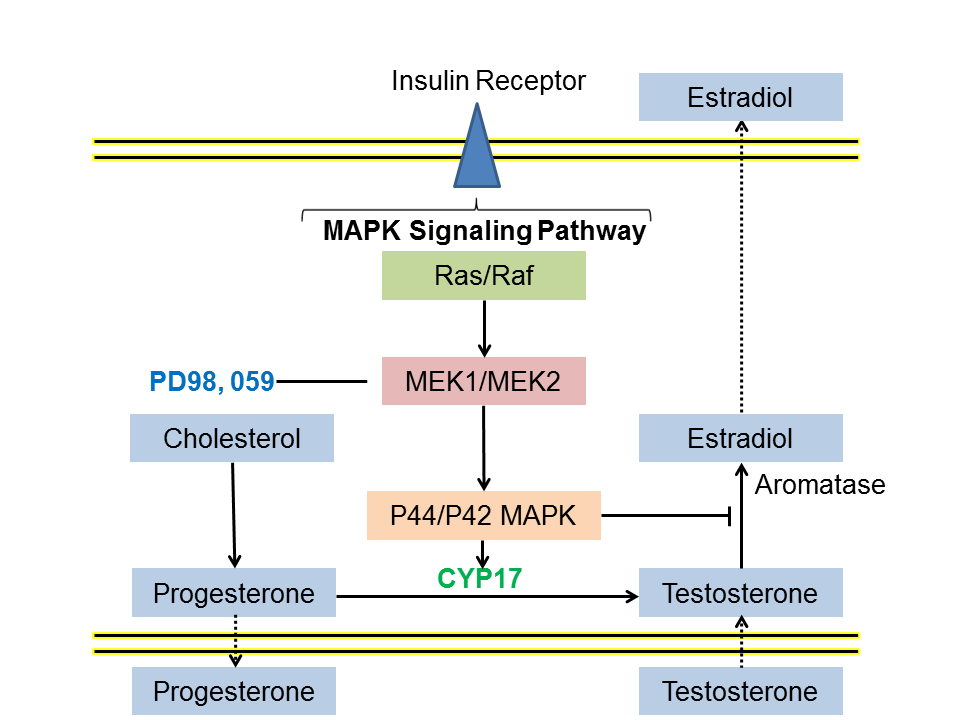

Supplement: S3 Fig — For normal GCs, P4 is converted from cholesterol followed by partial conversion into T through catalyzation of CYP17. External T is transferred into GC and converted into E2 along with internal T, which is later secreted through catalyzation of aromatase. For the GC IR model, up regulation of the p44/42 MAPK signaling pathway leads to the elevation of the CYP17 level while reducing T intake, resulting in an increased concentration of T. Inhibition of the p44/42 MAPK signaling pathway by PD98059 results in the down-regulation of CYP17. (TIF) [file pone.0188029.s003.tif]
